# Immune Modulation by Androgen Deprivation and Radiation Therapy: Implications for Prostate Cancer Immunotherapy

**DOI:** 10.3390/cancers9020013

**Published:** 2017-01-27

**Authors:** Jennifer L. Kalina, David S. Neilson, Alexandra P. Comber, Jennifer M. Rauw, Abraham S. Alexander, Joanna Vergidis, Julian J. Lum

**Affiliations:** 1 Trev and Joyce Deeley Research Centre, British Columbia Cancer Agency, Victoria, BC V8R 6V5, Canada; Jennifer.Kalina@bccancer.bc.ca (J.L.K.); dneilson@bccrc.ca (D.S.N.); acomber@bccrc.ca (A.P.C.); 2 Department of Biochemistry & Microbiology, University of Victoria, Victoria, BC V8P 5C2, Canada; 3 British Columbia Cancer Agency, Victoria, BC V8R 6V5, Canada; Jennifer.Rauw@bccancer.bc.ca (J.M.R.); AAlexander3@bccancer.bc.ca (A.S.A.); JVergidis@bccancer.bc.ca (J.V.); 4 Department of Medicine, University of British Columbia, Vancouver, BC V5Z 1M9, Canada; 5 Department of Surgery, University of British Columbia, Vancouver, BC V5Z 1M9, Canada

**Keywords:** androgen deprivation therapy, radiation therapy, immunotherapy, prostate cancer, cancer vaccines, checkpoint inhibitors

## Abstract

Prostate cancer patients often receive androgen deprivation therapy (ADT) in combination with radiation therapy (RT). Recent evidence suggests that both ADT and RT have immune modulatory properties. First, ADT can cause infiltration of lymphocytes into the prostate, although it remains unclear whether the influx of lymphocytes is beneficial, particularly with the advent of new classes of androgen blockers. Second, in rare cases, radiation can elicit immune responses that mediate regression of metastatic lesions lying outside the field of radiation, a phenomenon known as the abscopal response. In light of these findings, there is emerging interest in exploiting any potential synergy between ADT, RT, and immunotherapy. Here, we provide a comprehensive review of the rationale behind combining immunotherapy with ADT and RT for the treatment of prostate cancer, including an examination of the current clinical trials that employ this combination. The reported outcomes of several trials demonstrate the promise of this combination strategy; however, further scrutiny is needed to elucidate how these standard therapies interact with immune modulators. In addition, we discuss the importance of synchronizing immune modulation relative to ADT and RT, and provide insight into elements that may impact the ability to achieve maximum synergy between these treatments.

## 1. Introduction

In the 1940s, prostate cancers (PCa) were found to have a unique dependence on androgens. This discovery led to the emergence of a new approach to treat PCa using androgen deprivation therapy (ADT) [1]. Moreover, there is marked improvement in tumor control when ADT is combined with radiation therapy (RT), particularly for localized disease [2]. For many years, ADT and RT were presumed to work through a direct cytotoxic action on tumors; however, recent studies have uncovered under-appreciated benefits of these treatments on the immune system. In this review, we discuss current strategies in ADT and RT, the role each plays during the development of anti-tumor immune responses and the rationale for combining these standard therapies with immune modulation. We also summarize the current state of early clinical trials involving combinatorial strategies with ADT, RT, and immunotherapy and highlight important considerations for future trial design.

## 2. Standard Treatment Options for Prostate Cancer

Standard and potentially curative treatment options for localized PCa are typically determined based on risk grouping [3,4,5]. In recent years, there has been a shift toward active surveillance for men with low risk disease given the low disease-specific mortality observed in recent randomized trials [6,7]; however, active treatment may still be appropriate for some patients, especially those of a younger age. On the other hand, active treatment is felt to improve outcomes for men with intermediate or high risk disease [6,8,9]. Standard options for intermediate risk disease include radical prostatectomy, brachytherapy, and external beam radiotherapy (EBRT), with, or without, ADT [10]. Typical options for high risk disease include surgery (with or without adjuvant RT), EBRT and ADT, or EBRT and ADT with brachytherapy boost [10]. In addition, prostate stereotactic ablative radiotherapy (SABR) is an emerging modality that appears effective [11,12], although additional studies are needed to validate these early but promising findings.

### 2.1. Androgen Receptor Signaling in Prostate Cancer

The androgen receptor (AR) is a nuclear hormone receptor activated by engagement of its ligands, testosterone and dihydrotestosterone (DHT). Ligand binding results in the displacement of heat shock proteins and exposes the AR nuclear localization signal. In the nucleus, the receptor dimerizes and binds to androgen response elements (AREs) in the promoter regions of target genes (e.g., PSA (prostate-specific antigen)) [13]. Additional co-regulatory proteins are recruited to facilitate transcription, leading to downstream cellular responses such as growth and survival [14]. Androgens and the AR are the main regulators of PCa cell growth. Thus, androgen ablation therapies repress transcription of AR target genes, which causes activation of tumor cell apoptosis and the eradication of a large fraction of androgen-dependent cancer cells [15].

PCa regression and disease stability will often occur with ADT; however, disease progression is inevitable in patients with metastatic disease at presentation. PCa growth despite adequate first line ADT (defined by a castrate serum-free testosterone level) is described as castration-resistant prostate cancer (CRPC). Mechanisms of PCa progression in the setting of ADT are multifactorial. Metastatic PCa is a heterogeneous mix of both androgen-dependent and androgen-independent malignant cells. Continuous treatment with ADT will remove the larger population of androgen-dependent cells but allows for the selective outgrowth of androgen-independent cells. In addition, reactivation of AR signaling has been identified as an important driver in androgen resistance [16]. The loss of response to ADT is associated with post-castration activation of the AR via mechanisms such as AR mutation, gene amplification, incomplete blockade of ligand-dependent AR activation, and aberrant AR co-regulator activity [14]. Therefore, the AR plays an important role in both the castrate-sensitive and castration-resistant setting.

### 2.2. Current Strategies for ADT

ADT can be accomplished with either bilateral orchiectomy or medical castration using either gonadotrophin releasing hormone (GnRH) agonists or antagonists. These approaches are often combined with first-generation anti-androgen therapies, such as flutamide, bicalutamide, and nilutamide, to achieve total androgen blockage by inhibiting the effects of androgen production from the adrenal gland. For metastatic castration-sensitive disease, ADT is the standard approach [17] and superior outcomes may be achieved if ADT is combined with docetaxel chemotherapy [18,19]. For most patients, ADT is initially effective; however, despite these efforts a significant proportion of patients ultimately experience disease recurrence and progression to castration-resistant PCa [20].

Since the introduction of docetaxel in 2004, treatment options for metastatic castration-resistant prostate cancer (mCRPC) have changed dramatically. Several newer agents have been developed for mCRPC that demonstrate enhanced overall survival when given in conjunction with continued ADT. These so called “next-generation” strategies include drugs that interfere with androgenic stimulation, such as abiraterone and enzalutamide, as well as chemotherapy (e.g., docetaxel and cabazitaxel). Abiraterone is an irreversible inhibitor of cytochrome P450 17A1, which impairs androgen-receptor signaling by depleting both adrenal and intra-tumoral androgens. Abiraterone is often administered in conjunction with prednisone to counteract side effects related to compensatory adrenocorticotropic hormone (ACTH) production. Indeed, abiraterone plus prednisone extended overall survival, compared to prednisone alone, in two landmark phase III studies involving both chemotherapy-naïve CRPC, as well as men previously treated with docetaxel [21,22]. Similarly, enzalutamide, a competitive inhibitor of the AR, was shown to prolong overall survival compared to placebo in both chemotherapy-naïve and docetaxel-treated CRPC [23,24]. Despite some improvement in survival, these strategies ultimately are not curative. Thus, there remains a need for more effective approaches to treat men with mCRPC. In addition, many of these newer approaches have not yet been systematically compared in randomized trials, leaving several unanswered questions regarding the optimal selection, sequencing, and combination with other therapies including RT and immune modulation. For the purposes of this review, we have collectively used the term ADT to describe both anti-androgen and androgen deprivation techniques. While each has distinct physiological mechanisms of action, the main goal of these interventions is to halt PCa growth by inhibiting the androgen-AR axis.

### 2.3. The Role of RT in Prostate Cancer

#### 2.3.1. Curative Treatment for Localized Disease

RT is well established as an effective and potentially curative treatment for localized PCa, either alone or in conjunction with ADT [10]. Prostate RT can be delivered using EBRT or brachytherapy. Modern EBRT is typically administered as intensity modulated radiotherapy (IMRT) or volumetric modulated arc therapy (VMAT), in which multiple beams or arcs, each varying in intensity, are used to deliver high doses to the prostate while minimizing exposure to normal tissues. IMRT has been shown to significantly reduce both gastrointestinal and genitourinary toxicity compared to 3D conformal radiotherapy [25]. Image guidance through the use of fiducial markers, cone beam CT, or ultrasound can also be applied to further reduce treatment-related morbidity [26,27]. These techniques allow for RT dose escalation, which has been shown to improve biochemical disease-free survival [28,29,30]. Brachytherapy, in which radioactive seeds are placed within the gland either permanently or temporarily, can also be used to deliver highly conformal radiation doses to the prostate with excellent outcomes [31]. For higher risk disease, EBRT is often combined with a brachytherapy boost, providing a form of dose escalation with high rates of biochemical disease control [32,33].

#### 2.3.2. Palliative Treatment

RT also plays a major role in the palliative management of PCa in the metastatic, recurrent, or castration-resistant setting. Palliative EBRT is effective for treating symptoms from painful bony metastases [34] and has utility in the treatment of malignant spinal cord compression [35]. RT can also be useful for palliation of a variety of other local symptoms such as hematuria, hemoptysis, or painful soft tissue masses. Palliative pelvic RT can be useful in maintaining local control for prostate masses with potential for bladder or rectal invasion. In the setting of metastatic PCa, there is also emerging evidence that radical treatment of the primary tumor may confer survival benefit when given along with ADT or chemotherapy [36,37,38]. Radium-223 is a recent addition to the armamentarium for mCRPC. This radionuclide is a systemic bone-targeted calcium mimetic that is incorporated into areas of high bone turnover, such as in osteoblastic metastases [39]. This short-range form of radiation is able to treat local PCa cells while preserving nearby normal tissue, such as bone marrow. The pivotal Alpharadin in Symptomatic Prostate Cancer (ALSYMPCA) trial demonstrated improvements in overall survival, freedom from symptomatic skeletal events, pain control, and quality of life for radium-223 over placebo [40]. These data have led to its consideration as a standard treatment option for CRPC.

#### 2.3.3. Stereotactic RT for Oligometastatic Disease

There has been recent interest in the potential role of aggressive metastasis-directed therapy in the setting of “oligometastatic” or “oligo-recurrent” PCa, defined as few (≤5) metastatic lesions [41]. It is hypothesized that metastatic cancers with a low number of lesions may represent a state of “restricted metastatic potential” [41]. If so, definitive therapy directed at identifiable metastases may improve survival or delay the need for further systemic therapies. Under this strategy, RT is delivered to all metastatic lesions using a highly accurate, conformal, image-guided technique called stereotactic radiotherapy (SABR). With this technique, large “ablative” doses of radiation are delivered in a small number of fractions (typically less than five). Early data suggests this approach yields excellent control of metastatic lesions with encouraging rates of distant-, biochemical-, ADT-free, and overall survival [41,42,43,44]. Although this approach appears promising, validation in randomized controlled trials is required.

### 2.4. Combined ADT and RT

The combination of ADT and RT has been shown to improve overall, metastasis-free, prostate cancer-specific, and biochemical survival over RT alone for patients with intermediate to high risk and locally advanced PCa in a number of large, randomized trials [2,45,46,47,48,49]. For intermediate risk disease, 4–6 months of ADT appears sufficient to provide survival benefit [50,51]. For high-risk and locally-advanced disease, the optimal ADT duration is unknown, but regimens ranging from 18 to 36 months all appear effective and are commonly utilized [52,53,54]. The RT component of the combination is critical, with randomized evidence of inferior overall, prostate cancer-specific, and metastasis-free survival with ADT alone in high risk disease [55,56,57]. Two recent trials have also demonstrated clinical benefit with the addition of ADT to salvage RT in the treatment of biochemical relapse after primary surgery [58,59].

The addition of ADT to RT appears to improve outcomes by enhancing both local and distant disease control. Mechanisms of synergy are poorly understood, but are likely mediated by the AR. There are a number of possible ways by which ADT and RT improve disease control. For example, emerging data suggests that ADT can act as a “radiosensitizer” by inhibiting the tumor cell’s ability to repair double-stranded DNA damage [60,61]. Milosevic et al. [62] demonstrated that ADT reduces intraprostatic hypoxia. Hypoxia is associated with poor local control and biochemical failure after RT [63,64]. There is also evidence that improved local and distant control could be mediated by permanent cell cycle arrest or apoptosis induced by combined treatment [65]. Finally, enhanced immune responses have also been implicated in the synergy between ADT and RT [66,67,68], a mechanism that could underlie both local and systemic disease control.

## 3. The Immune Landscape in Prostate Cancer

There is a general consensus that the presence of tumor infiltrating lymphocytes (TIL) is associated with better patient outcomes; however, TIL responses are often weak or absent in PCa [69,70]. This might be due to the prostate gland itself, which has traditionally been considered an immunologically privileged site due to the lack of afferent lymphatics and the immunosuppressive properties of semen [71]. Despite this, when T cell infiltrates are present, they are often found at a lower density than in adjacent normal or hyperplastic regions, suggesting a non-permissive or inhospitable tumor microenvironment [69]. Instead, prostate tumors commonly contain elevated levels of CD4+ and CD8+ T cells with regulatory phenotypes (e.g., expression of CD25, FoxP3, CITR, ICOS) [72,73,74]. In addition, the frequency of infiltrating CD4+ T regulatory (T reg) cells is greater than what is often observed in classically immunogenic tumors, such as melanoma. Consistent with these findings, the prognostic significance of TIL in PCa remains controversial. Some studies show that TIL are associated with improved survival [75,76,77], while others describe no prognostic significance [71,77,78,79] or even negative associations with clinical outcome [76,77,80,81]. In general, it appears that PCa may not follow the trends related to the benefits of TIL as in other tumor types, likely because these TIL may be skewed towards more immunosuppressive phenotypes [72,73,82]. A tumor’s “immunological status” is of great importance when considering how to best enhance these responses with immunotherapies. In the next section, we consider some of the driving mechanisms that may play crucial roles in dictating how the immune system responds to PCa.

## 4. Mechanisms of Tumor Immune Evasion

### 4.1. Immune Camouflage

Continuous pressure from the immune system provides a selective mechanism for tumor evolution and immune evasion. For example, many PCa display abnormalities in Major Histocompatibility (MHC) Class I antigen processing machinery, including low levels of surface MHC [83]. In addition, compared to other solid tumors, PCa have a relatively low mutational load [84] which may render them less responsive to checkpoint therapies that rely on pre-existing antigen-specific T cells for their efficacy.

### 4.2. Immune Checkpoints

T cell activation is regulated by an intrinsic negative feedback loop involving the B7H family receptors and ligands. This is illustrated by two widely studied pathways which have become focal points for current approaches utilizing the immune system for cancer treatment. The first identified was CTL-associated antigen-4 (CTLA-4), which competes with CD28 for binding of co-stimulatory molecules (CD80/86) on antigen presenting cells, resulting in suppression of T cell activation [85]. Second, programmed cell death-1 (PD-1) is expressed on activated T cells, while its ligands, PD-L1 and PD-L2, are widely expressed in many non-lymphoid tissues, including tumors [85,86,87]. Engagement of PD-1 by its ligand induces a state of T cell exhaustion characterized by suppression of effector cytokine production and reduced proliferative capacity [86]. Physiologically, this system is crucial for preventing T cell auto-reactivity; however, tumor cells have co-opted these pathways to subvert cytolysis by host T cells. Indeed, studies suggest over 50% of primary PCa express moderate to high levels of PD-L1, which is associated with reduced biochemical recurrence-free survival after radical prostatectomy [88]. The efficacy of PD-1 and PD-L1 blocking antibodies to relieve this suppression and reinvigorate anti-tumor immune responses in PCa remains unclear, although one recent clinical trial suggests it may be beneficial for a subset of patients [89].

### 4.3. Cell-Mediated Immunosuppression

Several tumor-associated immune cell subsets have been identified as key players facilitating immunosuppression and tumor progression in PCa, including T regs, myeloid-derived suppressor cells (MDSC), tumor-associated macrophages (TAMs), and select B cell subsets. T regs are best described phenotypically as CD4+CD25+FoxP3+ T cells. This TIL subset has a classical role in mediating protection from autoimmunity and promoting tolerance via several immunosuppressive mechanisms [90]. It is, therefore, not surprising that T regs are enriched in both the tumor and peripheral blood of PCa patients [82,91]. Prostate tumors may also recruit MDSC, a heterogeneous population of immature myeloid cells and myeloid precursors. These cells actively suppress T cell responses within the tumor, and increased frequencies of circulatory MDSC in PCa patients has been shown to correlate with negative prognostic indicators, including elevated PSA levels and reduced overall survival [91]. Mononuclear MDSC can also differentiate into TAMs. Tissue resident macrophages adopt a spectrum of phenotypically diverse activation states in response to the different signalling cues within the tissue microenvironment. These activation states either support inflammation (M1-like), or suppress adaptive immune responses and promote tissue repair during the resolution of an immune response (M2-like) [92]. Within prostate tumors, TAMs are more commonly polarized toward a M2-like phenotype and promote angiogenesis, progression, and metastasis [92,93]. Not surprisingly, TAM infiltration has been shown to correlate with several clinicopathologic indicators including serum PSA, Gleason score and clinical T stage [94]. Finally, there is emerging evidence highlighting a role for B cells in the inhibition of anti-tumor CTL responses in PCa. A recent report identified a class of immunosuppressive tumor-infiltrating IgA+ plasmocytes in both human and murine PCa [95]. These cells expressed IL-10 and PD-L1, and mediated resistance to immunogenic chemotherapy by suppressing anti-tumor CTL. In addition, B cell-derived lymphotoxin was shown to be important for the development of castration-resistant disease in the Transgenic Adenocarcimoa of the Mouse Prostate (TRAMP) model of PCa [96]. This information is unexpected given the association between tumor-infiltrating B cells and favorable prognosis in other settings such as ovarian cancer [97].

### 4.4. Suppression of Antigen Presentation and T Cell Priming

Prostate tumors may also interfere with the earliest stages of T cell activation by causing priming of suboptimal T helper 2 (T_H_2) or T helper 17 (T_H_17)-type immune responses. Secretion of soluble mediators by prostate tumors (e.g., TGFβ IL-10, IL-6, COX-2, iNOS) interfere with dendritic cell (DC) maturation in a manner that inhibits strong T_H_1-type immune responses and can lead to induction of antigen-specific T cell anergy and outgrowth of T regs. In addition, tumor-derived soluble mediators (e.g., IL-10, COX-1/2, VEGF, GM-CSF, IL-1β) can alter DC differentiation to preclude development of cells with antigen-presentation function and instead skew differentiation of DC precursors into immunosuppressive TAMs and MDSC [98,99]. Lastly, PCa possess an additional unique mode of immunosuppression mediated by secretory PSA, which has been shown to inhibit generation and maturation of DC in vitro and suppress their ability to induce T cell proliferation [100].

Overall, prostate tumors create multiple barriers to achieving a successful, active, anti-tumor adaptive immune response. These features provide a unique opportunity to use immunotherapy as a means to overcome these negative regulatory networks in PCa.

## 5. The Role of ADT in Modulation of Immune Responses

Due to the inherent dependency of prostate tumors on the AR, the primary anti-tumor effect of ADT is a result of directly inducing tumor cell apoptosis. However, emerging evidence suggests that ADT may also indirectly lead to the priming of tumor-specific adaptive immune responses [101]. Although ADT may elicit these dual benefits on both the tumor and the immune system, patients treated with ADT, particularly those with more disease burden, often experience biochemical relapse. As discussed below, if ADT initially supports anti-tumor T_H_1-type responses, these are likely short-lived and eventually an immunosuppressive TIL landscape predominates. In these cases, it may be beneficial to provide additional stimuli, such as RT or immunotherapy, to promote skewing towards more favourable and durable anti-tumor immune responses.

New data has highlighted the importance of AR signalling in immune regulation. For example, conditional knockout of the AR in B and T cells improves lymphocyte development and activation, while castration causes thymic enlargement and increased levels of peripheral immature B cells and naive T cells [102]. This implies that androgens have a negative regulatory effect on lymphocyte development and activity. Indeed, androgens have been shown to directly inhibit T_H_1 differentiation [68]. Alternatively, there may also be a prostate-dependent effect on TIL after ADT. As demonstrated in animal models, castration is able to mitigate tolerance to prostate antigens and cause an influx of prostate-infiltrating lymphocytes that are, at least transiently, T_H_1-biased [103,104]. Therefore, it appears that the androgen-AR axis can have a profound suppressive effect on the behaviour of various lymphocyte subsets. It is not surprising, then, that ADT has a positive influence on anti-tumor immune responses; however, it prompts to question why PCa patients treated with standard ADT fail to develop effective immune responses and eventually experience tumor relapse. In one clinical report, ADT promoted strong adaptive anti-tumor T and B cell responses; however, peripheral T_H_1 and T_H_17 effector memory subsets were diminished after two years of therapy [101]. Thus, it appears that these beneficial responses may be short-lived, or even blunted, by concomitant changes in other lymphocyte subsets. For instance, castration was shown to cause induction of strong anti-tumor CD8+ T cell responses but these changes were accompanied by a concomitant increase in CD4+CD25+FoxP3+ T regs [105]. A similar report by Sorrentino et al. [78] showed that patients treated with hormone therapy prior to radical prostatectomy had increased levels of TIL with both cytotoxic and regulatory T cell phenotypes. Furthermore, it was recently shown that ADT stimulates tumor cells to produce macrophage colony stimulating factor-1 (M-CSF1), leading to increased TAM infiltrates in both PCa patients and tumor-bearing mice [106]. Finally, new evidence shows that the type of ADT can be a determining factor in how adaptive immune responses change following androgen ablation. In a recent preclinical report by Pu et al. [107], the authors conducted a direct comparison between surgical androgen depletion, a LHRH-analogue, and an AR antagonist (flutamide). First, neither surgical nor LHRH-analogue ADT resulted in inhibitory effects on T cell responses. In contrast, flutamide interfered with initial T cell priming, impaired efficacy of anti-PD-L1 therapy, and led to earlier tumor relapse. Thus, it appears that the specific mode of androgen suppression has important implications for downstream immunological effects. Whether more advanced AR blocking agents (e.g., enzalutamide and abiraterone) share these immunosuppressive properties is a critical question that needs to be resolved. Next, we discuss the concepts related to RT-induced immune activation and how RT, in combination with ADT, may be beneficial for anti-tumor immunity.

## 6. How RT Improves Tumor Immunogenicity

### 6.1. RT Promotes Immunogenic Cell Death and Antigen Presentation

Tumor cell death is a prime source of antigens for uptake by DC. Indeed, a secondary response to RT is the release of endogenous tumor antigens via apoptotic or necrotic cell death, which are captured by DC, processed, and presented to CD4+ and CD8+ T cells. A large body of preclinical evidence and several clinical case reports support the notion that RT can favor anti-tumor immune activation. First, RT has been shown to increase tumor MHC class I expression in a dose-dependent manner both in vitro and in vivo [108]. RT also causes release of danger-associated molecular patterns (DAMPs) from stressed or dying tumor cells, including pro-inflammatory cytokines (e.g., TNFα, IFNγ, IL-1α/β) and other immune-stimulatory factors (e.g., ATP, HMGB1, cyclic dinucleotide, calreticulin) in what has been described as ‘immunogenic cell death’ [109,110,111]. For these reasons, RT is thought to act as an “in situ tumor cell vaccine”, whereby CD4+ and CD8+ T cell responses are generated through antigen release and acquisition by DC in the presence of the appropriate stimuli.

### 6.2. RT Disrupts the Balance Between Pro-Inflammatory and Immunosuppressive Soluble Factors

Numerous studies have demonstrated that RT leads to increased local production of soluble factors that promote anti-tumor immunity by enhancing activation of local innate immune cells and recruitment of tumor-reactive T cells (e.g., CXCL16, IL-1α/β, IFNγ, and TNFα) [109,112,113]. Paradoxically, these factors may also play a role in tumor progression. For example, IL-1α and IL-1β promote angiogenesis and invasiveness in some cancer models [114], and other studies have found a link between elevated plasma levels of TNF and negative clinical outcomes [115]. It should be pointed out that RT has purported roles in immune suppression, including RT-induced production of tumor M-CSF1. In principle, this may increase the levels of circulating MDSC [116], adding complexity to how RT can alter the immune response in PCa. Furthermore, RT has been shown to promote activation of TGF-β1. This cytokine, while classically known for its immunoregulatory properties [117], induces CD103 expression on activated T cells, which facilitates tumor infiltration and recognition. Indeed CD103+ TIL are associated with favorable prognosis in some settings [118,119,120]. Whether RT leads to increased CD103+ TIL in PCa is not yet known; however, these data show that RT-induced production of soluble factors can have both complementary and opposing effects which may have implications for subsequent immune modulation.

## 7. Can ADT and RT Synergize with Immunotherapy?

**Figure 1:**
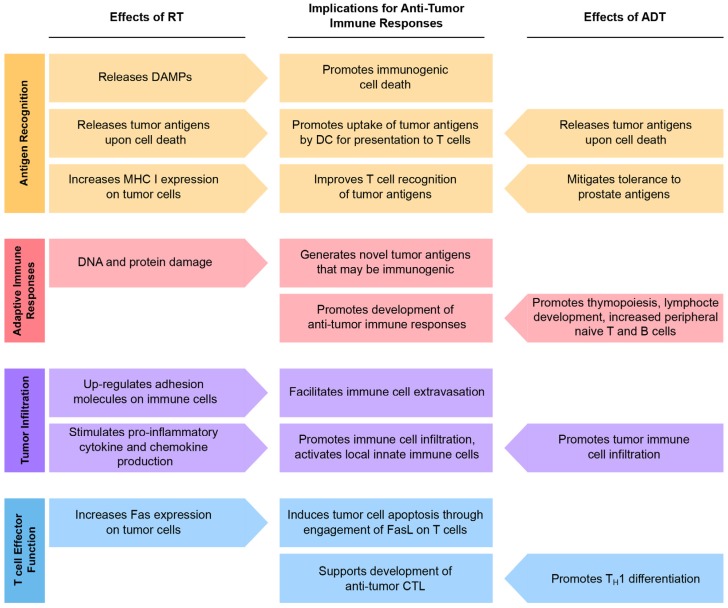
Cellular and molecular effects of ADT and RT as they relate to the development of anti-tumor immunity.

ADT and RT can independently enhance tumor immunity by modulating both local and systemic molecular and cellular responses (summarized in Figure 1). If this combination is superior to either treatment alone, why then do we not observe more exceptional outcomes in patients treated with these modalities? In fact, a large proportion of patients treated with ADT and RT eventually progress to castration-resistant disease. While there are many possible reasons for this, we speculate that both the class of ADT and the sensitivity of tumors to radiation are crucial parameters that determine clinical responses. Furthermore, given the dual nature of some of the effects of ADT and RT discussed above, the temporal sequence of these treatments relative to each other could have either synergistic or antagonistic consequences on local and systemic immune responses. Therefore, any attempts to elicit robust responses with immune modulatory agents must take careful consideration of the treatment scheme (i.e., type, dose, duration, and timing). Another concern is when peak tumor cell death and release of “immune signals” (e.g., DAMPs, tumor antigens) occurs as a result of ADT or RT, as these are transient processes that need to be precisely timed with immune modulation. Given what we know, one simple way to think about combinational treatment is that ADT and RT may prime anti-tumor T cell responses but full conversion to effector activity may require additional immunotherapeutic intervention.

Preclinical studies have demonstrated that castration may augment vaccine-induced immune responses [121]. A notable case report of a patient with mCRPC who achieved a complete and durable PSA decline after treatment with ADT and sipuleucel-T highlights the potential for combination strategies in advanced human PCa [122]. This patient, having progressed on enzalutamide and LHRH-agonist therapy, received sipuleucel-T and six months later experienced a drastic PSA decline that remained undetectable thereafter. The authors point out that such drastic PSA reductions after sipuleucel-T are rare, and the delayed onset of the response implies an immune-mediated mechanism. Furthermore, anecdotal reports of patients experiencing reductions in untreated metastases after receiving RT to a single lesion, a phenomenon known as the abscopal response, are believed to be systemic anti-tumor immune responses triggered by RT [123,124,125,126,127,128]. Several case reports of abscopal responses in patients treated with RT and immunotherapy suggest that the paucity of this phenomenon might be improved by combination strategies [128,129,130]. Indeed, this has been repeatedly observed in pre-clinical models [131,132,133,134] and affirmed by a small retrospective cohort study of sequential checkpoint blockade (ipilimumab) and RT in melanoma, where abscopal responses were observed in 52% of patients analysed (n = 21) and were correlated with improvements in overall survival [130]. To date, there have been no known accounts of abscopal responses in metastatic PCa; however, there is some clinical evidence of induction of anti-tumor immune responses after RT. Nesslinger et al. [135] found that 14% of patients who received EBRT and 25% of those treated with brachytherapy developed tumor-specific antibody responses, while none were detected in patients who underwent radical prostatectomy. In addition, two new reports suggest a survival benefit for patients with metastatic disease treated with ADT and prostate RT [38,136]. This emerging evidence in favor of local therapy in the metastatic setting has led to the initiation of several clinical trials [137,138,139], providing a possible opportunity to evaluate abscopal responses in metastatic PCa.

## 8. Current Status of Early Clinical Trials of Immunotherapy in Combination with ADT and RT

Clinical trials with several different classes of immune modulators are now being intensely pursued in PCa. In 2010, the FDA approved the use of sipuleucel-T (Provenge^®^, Dendreon, Seattle, WA, USA) as the first therapeutic vaccine for minimally symptomatic mCRPC. Several trials have tested ipilimumab (Yervoy^TM^, Bristol-Myers Squibb, New York, NY, USA) and more recently, ongoing trials with pembrolizumab (KEYTRUDA^®^, Merck Sharp & Dohme Corp., Kenilworth, NJ, USA) and nivolumab (Opdivo^®^, Bristol-Myers Squibb, New York, NY, USA) as checkpoint blockade immunotherapy in PCa. While these advances have been encouraging, in many cases immune modulation alone fails to provide superior improvements in survival compared to conventional anti-cancer therapies [140,141]. Thus, it appears that the many barriers to achieving anti-tumor immunity continue to hinder the success of current immunotherapies and more effective alternatives are clearly needed. Here, we summarize the findings of early clinical trials evaluating ADT and RT in combination with different immunotherapy modalities, with the presumption that the direct cytotoxic and immune-stimulating properties of ADT and RT outlined above may synergize with immunotherapy and augment the efficacy of either modality on its own. One key consideration is that the majority of clinical trials for advanced PCa employ ADT as the standard of care, and were not designed to evaluate the effect that ADT may have on clinical or immunological outcomes. However, there is an appreciation that ADT can alter the course of immune responses and new trials are being planned with this consideration in mind. Importantly, the class of ADT will likely be crucial, but this question has not yet been evaluated. In the studies discussed here, some of the details regarding ADT are not made available, although most patients remain on continuous androgen suppressive therapy for the duration of the trial unless specifically stated otherwise. A complete list of the current and ongoing clinical trials combining ADT, RT, and immunotherapy in PCa are provided in Table 1.

### 8.1. Vaccines

Therapeutic vaccination against tumor associated antigens (TAA) has been explored as a means to promote DC activation and antigen presentation to T cells in cases where there may be a lack of available antigens or necessary maturation signals. Cancer vaccines come in many forms, including, but not limited, to direct DNA, mRNA, or peptide injection, injection of autologous TAA-expressing DC, or DC co-cultured with tumor cell lysates, and injection of TAA- or cytokine-expressing recombinant viral vectors. This section will focus on select vaccine trials involving both ADT and RT.

The first approved immunotherapy for PCa, sipuleucel-T, involves infusion of a patient’s autologous DC that have been pre-loaded with a recombinant fusion protein consisting of a known prostate TAA, prostatic acid phosphatase (PAP), fused with granulocyte-macrophage colony-stimulating factor (GM-CSF). In the original phase III trial, sipuleucel-T imparted a moderate improvement in overall survival compared to the placebo (25.8 months vs. 21.7 months) [140]. This modest, but encouraging finding has prompted strategies of sipuleucel-T with ADT and RT, and the results of several ongoing clinical trials are pending [142,143,144,145]. In a pilot study of intraprostatic DC injection, patients initiated fractionated EBRT while remaining on androgen-suppressive therapy (GnRH-agonist and bicalutamide) [146]. Autologous DC were injected following fractions 5, 15, and 25 of EBRT, allowing approximately 72 h before the next radiation dose. After 25 fractions (45 Gy total), patients proceeded to brachytherapy. Two patients had induction of prostate antigen-specific T cell responses after the initiation of treatment. Conversely, one patient had pre-existing T cell responses to PSA, PSMA, and Her2/neu that were diminished at later time points. Although this treatment approach was determined feasible, the small sample size (n = 5) precluded conclusions regarding clinical benefit. Larger studies are needed to further uncover the optimal coordination of DC-based immunotherapy with the peak of RT-induced tumor apoptosis and inflammatory responses.

The use of three co-stimulatory molecules (B7.1, ICAM-1, and LFA-3) in a carcinoembryonic antigen (CEA) recombinant viral vaccine (called TRICOM) was shown to enhance T cell proliferation and confer an overall survival advantage in CEA+ tumor-bearing mice compared to vaccination with only one or none of these molecules [147]. Based on these findings, several phase I and II clinical trials have been launched using recombinant viral and TRICOM-based vaccines targeting PSA, CEA, and mucin-1 (MUC-1) in various cancer settings [148,149,150,151,152]. Currently, a handful of proof-of-concept studies have been completed that assessed safety recombinant viral-B7.1- or TRICOM-based vaccines against PSA in conjunction with standard ADT and RT in PCa [153,154,155]. A randomized phase II trial found that patients treated with PSA-TRICOM and ^135^Sm-EDTMP had increased levels of PSA-specific T cells and lower levels of circulating MDSC subsets compared to patients in the ^135^Sm-EDTMP alone arm at 60 days post-therapy [155]. In this same study, >30% PSA declines were only observed in the vaccine arm. While no statistical difference in overall survival was observed, patients receiving ^135^Sm-EDTMP and PSA-TRICOM had progression-free survival (PFS) more than twice that of those receiving ^135^Sm-EDTMP alone (3.7 months vs. 1.7 months, respectively).

In another phase II study, standard EBRT and ADT were combined with a similar vaccine strategy against PSA using rV-B7.1, IL-2 and GM-CSF [153]. Here, 13/17 patients treated with RT plus the vaccine regimen had at least three-fold increases in circulating PSA-specific T cells post-vaccination, while patients in the RT alone control arm had no detectable increase in such T cells (*p* < 0.0005) [153]. In two of the responding patients, the increase in PSA-specific T cells was observed following completion of RT, suggesting that RT enhanced immune responses to PSA in some cases. Conversely, eight patients had increases in PSA-specific T cells post-vaccination that then decreased following RT; however, these levels recovered with subsequent vaccine boosts in four patients. This study also noted evidence of epitope spreading in 6/8 patients evaluated, as indicated by the appearance of new responses to other known PCa associated antigens (PMSA, PAP, PSCA, and MUC-1) after vaccination but before the initiation of RT. Finally, while there was no significant difference in PSA-specific T cell responses between patients in the vaccine arm who did or did not receive ADT (3/17), there were too few patients to draw any conclusions regarding the immunological effects of ADT in this setting.

These early data suggest that ADT and RT in combination with recombinant viral vaccines is feasible, although it is not clear to what extent RT contributes to, or antagonizes, anti-tumor immunity induced by vaccination in these studies. In the case of the latter, it appears it may be possible to recover any immunosuppressive effects of RT with subsequent immune modulation.

### 8.2. Checkpoint Blockade

Checkpoint blockade immunotherapies have contributed to one of the most significant improvements in cancer therapeutics to date. Currently, three indications have been approved by the FDA for treating melanoma, NSCLC, and renal cell carcinoma: ipilimumab, pembrolizumab, and nivolumab. In general terms, this class of immune modulators comprises antibodies that block the interactions between the B7H receptor-ligand family of surface co-stimulatory molecules. In either case, these agents alleviate T cell suppression during activation and effector stages. There are two general classes of approved checkpoint blockade inhibitors: those that target CTLA-4, and those that target the PD-1/PD-L1/L2 pathway. The principal idea behind checkpoint blockade in combination with RT is that tumor cell killing by RT acts as an in situ vaccine that can help release TAA and pro-inflammatory factors that promote priming of systemic anti-tumor T cell responses. These responses are then enhanced by checkpoint blockade, which minimizes ongoing suppression from T cell engagement with tumors and surrounding suppressor cells. This concept has been successfully demonstrated in numerous preclinical tumor models but here we will focus on human trials that have attempted to recapitulate these results.

A randomized, double-blind phase III clinical trial comparing ipilimumab monotherapy (n = 399) to placebo (n = 400) in mCRPC patients having undergone a single fraction of RT (8 Gy) and prior docetaxel did not reach statistical significance with its primary endpoint of overall survival, but found that ipilimumab was associated with reductions in PSA and slight improvements in PFS (median 4.0 months vs. 3.1 months PFS) [156]. Despite this study not meeting its primary endpoint, the authors suggest that these signs of anti-tumor activity warrant further investigation of ipilimumab in PCa, especially amongst a less advanced population that have not received prior chemotherapy.

Immunotherapy targeting the PD-1 axis is seeing unprecedented responses, particularly in the settings of melanoma and NSCLC [157,158,159,160]. Notably, patients treated with pembrolizumab or nivolumab have significant improvements in overall survival and PFS, and experience reduced toxic side effects compared to treatment with ipilumumab or other standard chemotherapy regimens [157,158,159,160,161]. Currently, several clinical trials of PD-1 blockade are ongoing in the PCa setting [162,163,164]. Early results from a phase II trial involving pembrolizumab in combination with enzalutamide reported complete PSA responses in 3/10 patients, two of which also experienced partial tumor reductions [89]. This latest report supports the continued examination of PD-1 blockade in PCa. Clinical trials evaluating safety and efficacy of anti-PD-1 immunotherapy in combination with ADT and RT are not yet underway.

## 9. Synchronization of Immunotherapy with ADT and RT

### 9.1. RT Dose and Fractionation

The ability of RT to induce an anti-tumor immune response depends on both the dose and fractionation scheme as well as the inherent properties of the tumor itself [165,166]. To date, the absolute dose required to elicit immune effects in a clinical setting is undefined and likely patient-dependent, although some evidence suggests the relative immunogenicity of a tumor positively correlates with increasing dose of radiation [108,112]. On the contrary, one study demonstrated that while increasing doses of single fraction RT correlated with increased tumor-reactive T cells, higher doses (e.g., 15 Gy) offset immune responses due to an elevation in T reg populations [166]. As a result, a more moderate dose (7.5 Gy) offered the most effective tumor control by instigating anti-tumor T cell responses while avoiding concomitant increases in T regs. In addition, one must consider how single-dose versus fractionated RT schemes affect immune modulation. Fractionation is routinely employed to permit recovery of normal tissue between treatments while targeting tumor cells during the most sensitive phases of the cell cycle. While single or hypofractionated high-dose RT schemes have been suggested to provide superior immune-mediated tumor control [167,168], using a fractionated scheme may help sustain pro-inflammatory cytokine production, thus opening a larger window of opportunity for synergy with immunotherapy [109]. For instance, one study demonstrated that fractionated (3 × 8 Gy and 5 × 6 Gy), but not single-dose (20 Gy) RT induced abscopal responses when combined with CTLA-4 blockade in a breast cancer model [133].

### 9.2. Timing

In general, RT promotes T cell priming through the release of tumor antigens and pro-inflammatory soluble mediators while ADT promotes lymphopoiesis, immune cell trafficking and tumor infiltration. Both strategies can be used in combination with immunotherapy to enhance these processes. Thus, maximum synergy may be achieved by precisely timing each intervention during the appropriate phase of a therapy-induced immune response. However, the timing of immune modulation is not straightforward and depends on many factors, such as the type of ADT, the RT strategy (i.e., type, dose, duration), and the immunotherapeutic agent being administered. For instance, in an animal model of colorectal cancer, anti-CTLA-4 immunotherapy was most effective when given prior to RT, while an OX40 agonist antibody was optimal when delivered one day after RT [177]. In another study, anti-PD-1 was only effective if administered concurrently, but not following fractionated RT [178]. On the other hand, it may be ideal to deliver intratumoral DC between fractionated RT cycles, taking into account the effects of RT on DC and their migration to tumor-draining lymph nodes [146,179]. The timing of immune modulation is further complicated by the tumor’s inherent susceptibility to ADT and RT. Indeed, radioresistance and androgen independence are characteristic of PCa [180,181]. One may need to consider a personalized approach first by identifying susceptible characteristics of an individual tumor, and second by devising an appropriate strategy that considers therapy synchronization. No doubt, there is clear evidence demonstrating the importance of coordinated therapy [177,178,182] and this concept is gaining recognition in the clinical setting [146,153]. However, there has yet to be developed standardized definitions and assays for quantitative clinical evaluation of therapy-induced immunologic effects. With this question in mind, new approaches to identify signatures of immunogenic cell death and model T cell trafficking are under development [137,183,184].

## 10. Concluding Remarks

ADT is not immunologically inert; however, many trials were not historically designed to consider the potential effects of ADT on subsequent immune modulation. In many cases ADT is administered at the discretion of the treating physician, however further published details (e.g., type and timing) are scarce. As we move into an era of cancer immunotherapy, the effects of ADT on the immune system and its impact on the success of emerging immunotherapies in PCa will require careful scrutiny in future trial designs. This is especially important in light of new information that certain classes of ADT may actually have negative immunological consequences [107]. Nonetheless, experience thus far, both in animal models and in the clinic, highlights the promise of ADT, RT and immunotherapy as a combination strategy, a prospect that awaits the results of upcoming phase III trials.

## Figures and Tables

**Table 1:** Current clinical trials involving immunotherapy in combination with ADT and RT in PCa (completed and in-progress).

ClinicalTrials.Gov Identifier	Phase	Immunotherapy	ADT	RT	Timing
NCT02107430 [169]	2	DCVAC/PCa	LHRH-a	Standard EBRT	DCVAC/PCa after RP and RT; Neo-adjuvant LHRH-a
PMC4241355 [146]	0	Dendritic Cell Vaccine	GnRH-a and Bicalutamide	EBRT (45 Gy in 25 fractions) and BT	ADT start 30–44 days before RT; intraprostatic DC injection after fractions 5, 15, and 25
NCT00323882 [170]	1, 2	Ipilimumab	Prior disease progression w/ADT	EBRT (8 Gy/lesion)	Prior ADT; Single dose RT to bone metastases <2 days before Ipilimumab
NCT00861614 [171]	3	Ipilimumab	Prior ADT	EBRT (8 Gy/lesion)	Prior ADT; Single dose RT to bone metastases <2 days before Ipilimumab or placebo
NCT01777802 [172]	0	Monitor Timing for Immune Modulation	Prior ADT	SBRT	Monitor for immune changes after RT
NCT01436968 [173]	3	ProstAtak™(AdV-tk)	6 months ADT	Standard EBRT	Two doses of ProstAtak™(AdV-tk) or placebo before RT, 3ird dose during RT; short term (6mo) ADT optional
NCT00005916 [174]	2	rV-PSA, rV-B7.1, GM-CSF and IL-2	Ongoing ADT allowed	Standard EBRT +/− BT	GM-CSF on days 1-4, rV-PSA/rV-B7.1 on day 2, low dose IL-2 on days 8–21 (repeat cycle every 28 days); RT after 3rd cycle
NCT00005916 [174]	2	rV-PSA, rV-B7.1, GM-CSF and IL-2	Ongoing ADT allowed	Standard EBRT +/− BT	GM-CSF on days 1–4, rV-PSA/rV-B7.1 on day 2, IL-2 on days 8–12 (repeat cycle every 28 days); RT between 4th and 6th cycle
NCT00450619 [175]	2	PROSTVAC-TRICOM	Ongoing ADT	^153^Sm-EDTMP	PROSTVAC-TRICOM on day 1, 15, 29, and every 28 days thereafter; ^153^Sm-EDTMP starting on day 8 and every 12 weeks thereafter
NCT01807065 [142]	2	Sipuleucel-T	Disease progression w/ADT	EBRT	RT in weeks 1–2 to a single metastasis, Sipuleucel-T on days 22, 36, and 50
NCT01818986 [143]	2	Sipuleucel-T	Ongoing ADT	SABR	Not specified
NCT02463799 [144]	2	Sipuleucel-T	Disease progression w/ADT	^223^Ra	^223^Ra every 4 weeks (6 cycles); Sipuleucel-T every 2 weeks starting on week 6 (3 cycles)
NCT02232230 [145]	2	Sipuleucel-T	Prior ADT	EBRT	RT to metastases 28 days prior to Sipuleucel-T
NCT01496131 [176]	2	Tecemotide (L-BLP25)	Goserelin	EBRT (54–72 Gy in 30–40 fractions)	Tecemotide and ADT start 2–3 months before starting RT

Abbreviations: luteinizing-hormone-releasing hormone analogues (LHRH-a); gonadotropin-releasing hormone agonist (GnRH-a); samarium-153-ethylenediamine tetramethylene phosphonic acid (^153^Sm-EDTMP); radium-223 (^223^Ra); recombinant Vaccinia (rV); prostate-specific antigen (PSA); stereotactic ablative radiotherapy (SABR); Interleukin-2 (IL-2); granulocyte macrophage colony stimulating factor (GM-CSF); external beam radiation therapy (EBRT); brachytherapy (BT); stereotactic body radiation therapy (SBRT); radical prostatectomy (RP).
